# The Impact of the COVID-19 Pandemic on Diagnosis of Skin Cancer Cases in North Cancer Alliance and Scotland

**DOI:** 10.7759/cureus.25019

**Published:** 2022-05-15

**Authors:** Stanislau Makaranka, Freya Scutt, Kaz Rahman

**Affiliations:** 1 Plastic Surgery, Aberdeen Royal Infirmary, Aberdeen, GBR

**Keywords:** covid-19 pandemic, scotland, public health care, non melanoma carcinomas, melanoma skin cancer

## Abstract

The coronavirus disease 2019 (COVID-19) pandemic has dramatically impacted healthcare provision in the UK and skin cancer services have had to adapt to ensure continuity of safe care. As we return to “normality” we reflect on lessons learned and the impact of the pandemic on skin cancer services.

We looked at data on Public Health Scotland Dashboard, which compiles data from 14 local health boards across Scotland, comparing melanoma and non-melanoma skin cancer diagnoses during the years 2020 and 2019 (pre-COVID-19 pandemic). We looked at skin cancer cases within the North Cancer Alliance (NCA) and all of Scotland.

Within the NCA, 518 cases of melanoma were diagnosed in 2019, compared to 429 in 2020. Within Scotland, 1950 cases of melanoma were diagnosed in 2019, compared to 1605 in 2020. In 2019, 5103 non-melanoma skin cancer cases were diagnosed in NCA, compared to 4071 in 2020. In Scotland, 21,626 non-melanoma skin cancer cases were diagnosed in 2019, compared to 16,193 in 2020.

The COVID-19 pandemic has had a significant impact on skin cancer care within the NCA and the whole of Scotland. A significantly lower number of melanoma and non-melanoma skin cancer cases have been diagnosed within the NCA and in Scotland in 2020 compared to 2019. The trend is similar between NCA and other areas of Scotland. We must raise awareness of melanoma and non-melanoma skin cancer to improve timely presentation of patients during a global health crisis and a multidisciplinary approach is needed to address this problem.

## Introduction

The North Cancer Alliance (NCA) is a collaboration of six NHS Boards in the north of Scotland, which serves a population base of 1.4 million people [1]. This represents 26% of Scotland’s total population, however covers 61% of Scotland’s land mass; the aim of the collaboration is to improve outcomes for patients diagnosed with cancer [1]. There are equivalent regional cancer networks in the west and south-east of Scotland, which are called WoSCAN and SCAN respectively. Following the onset of the coronavirus disease 2019 (COVID-19) pandemic, the North Cancer Alliance and the other two regional cancer networks highlighted concerns about a reduction in the number of recorded skin cancer diagnoses, despite the number of diagnoses increasing year-on-year pre-pandemic. We set out to investigate the impact of the COVID-19 pandemic on diagnosis of skin cancer, hypothesising that the pandemic has had a detrimental effect on the presentation and diagnosis of potential patients with melanoma and non-melanoma skin cancer.

## Materials and methods

Study design

We looked at data on Public Health Scotland Dashboard, which compiles data from 14 local health boards across Scotland, comparing melanoma and non-melanoma skin cancer diagnoses during the years 2020 and 2019 (pre-COVID-19 pandemic). We looked at skin cancer cases diagnosed within the NCA and all of Scotland. The data is collected by the Scottish Cancer Registrations Team, who use an electronic registration system to collect information from a number of sources [2]. These include hospital patient administration systems, screening datasets, death records from National Records Scotland (NRS), private hospitals and community prescribing [2]. These records are updated on a monthly basis and are freely accessible to anyone online.

Inclusion and exclusion criteria

For malignant melanoma we looked at all cases confirmed on a pathological specimen during the years 2019 and 2020. Patients of all ages and gender were included. Initially we looked at regional cancer networks (NCA, SCAN and WoSCAN) individually and then at the whole of Scotland. We excluded all non-melanoma skin cancers and other cancers diagnosed during the same time period.

We then looked at non-melanoma skin cancer diagnoses confirmed on a pathological specimen during the years of 2019 and 2020. Inclusion criteria comprised patients of all ages or gender and all melanoma and other skin cancers were excluded. Again, we initially looked at the above information for the regional cancer networks (NCA, SCAN, WoSCAN) individually and then at the whole of Scotland.

Following this we looked at all malignant neoplasms in patients of all ages or gender in the whole of Scotland during 2019 and 2020. We excluded cancers diagnosed during other time periods.

Data collection

The data we collected is accessible to anyone online at Public Health Scotland Dashboard. On the "cancer" tab of the dashboard we selected a "geography level" from the available drop-down options. The geography levels we chose were the three regional cancer networks and "Scotland". After that we used the "select all or specific cancer type" drop-down option to choose either melanoma or non-melanoma skin cancer subtypes, followed by all malignant neoplasms. Once the suitable drop-down options were chosen we clicked on the "download data" tab to download graphs showing the information of interest.

Statistical analysis

The statistical analysis involved looking at the number of total cancer diagnoses (broken down by cancer subtype and geographical area) for the years 2019 and 2020 and then calculating the percentage difference between 2020 and 2019 (pre-pandemic). When looking at our graphs we paid particular focus to the time shortly after the first lockdown, when a divergence in the number of cancer cases diagnosed was observed.

## Results

**Figure 1:**
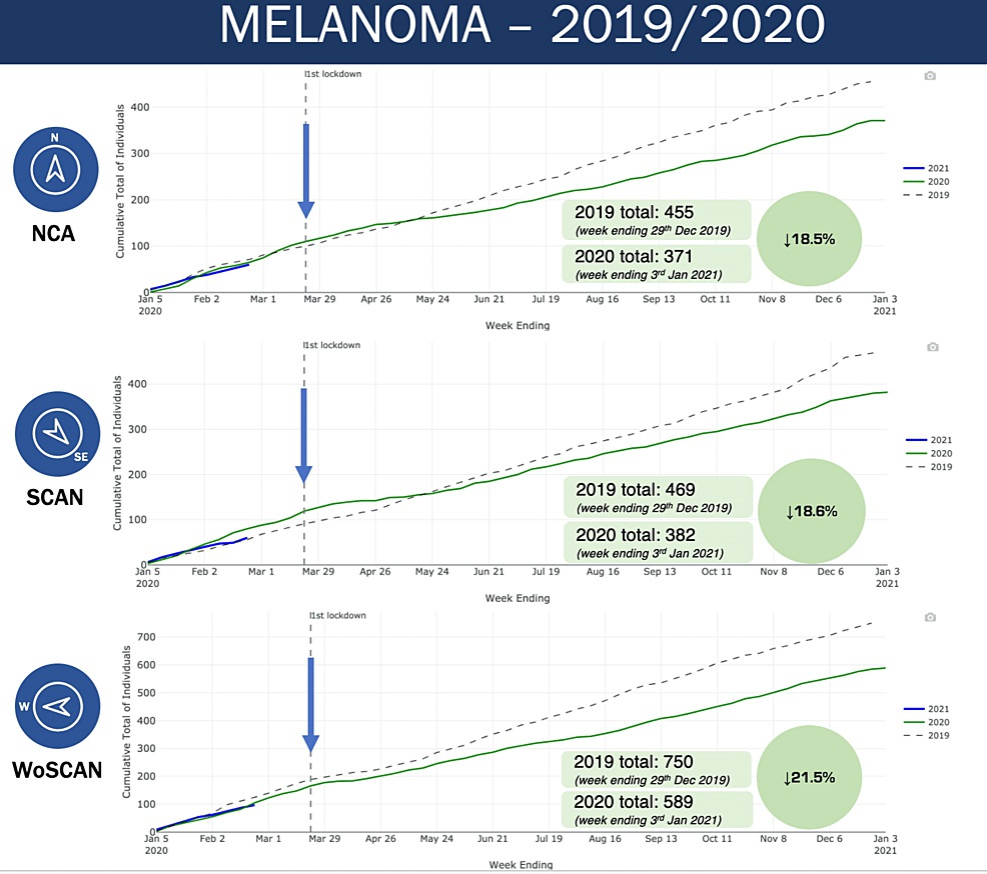
Melanoma diagnoses in NCA, SCAN and WoSCAN networks NCA: North Cancer Alliance, SCAN: South East Scotland Cancer Network, WoSCAN: West of Scotland Cancer Network

The initial data we looked at was for the number of melanoma diagnoses in the North Cancer Alliance as well as SCAN and WoSCAN networks in 2019 and 2020 (Figure 1). On the graph the number of melanoma diagnoses is initially similar in 2019 and 2020, however shortly after the first lockdown there is a divergence, with the number of diagnoses plummeting in early 2020 which continues through the rest of 2020. This pattern is evident for all three regional cancer networks. This resulted in an 18.5% reduction in the number of melanoma diagnoses in the NCA, and 18.6% and 21.5% reduction for the SCAN and WoSCAN networks respectively. This is despite us knowing, from data on Public Health Scotland Dashboard, that melanoma diagnoses have been increasing year on year pre-pandemic.

**Figure 2:**
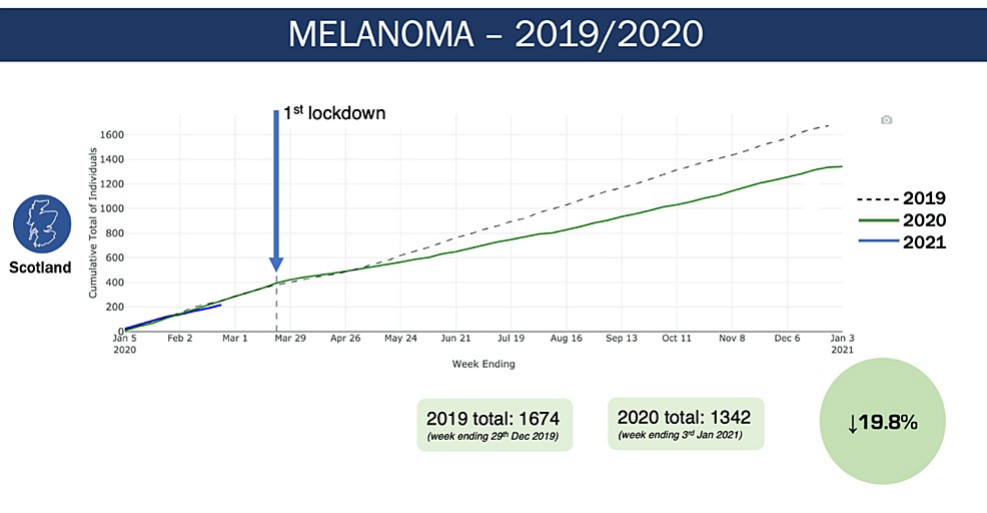
Melanoma diagnoses in all of Scotland

When looking at the number of melanoma diagnoses in all of Scotland (Figure 2), we can see the same pattern as described above, with a divergence in the number of diagnoses shortly after the first lockdown. This resulted in a 19.8% reduction in the number of diagnoses in 2020 when compared to 2019.

**Figure 3:**
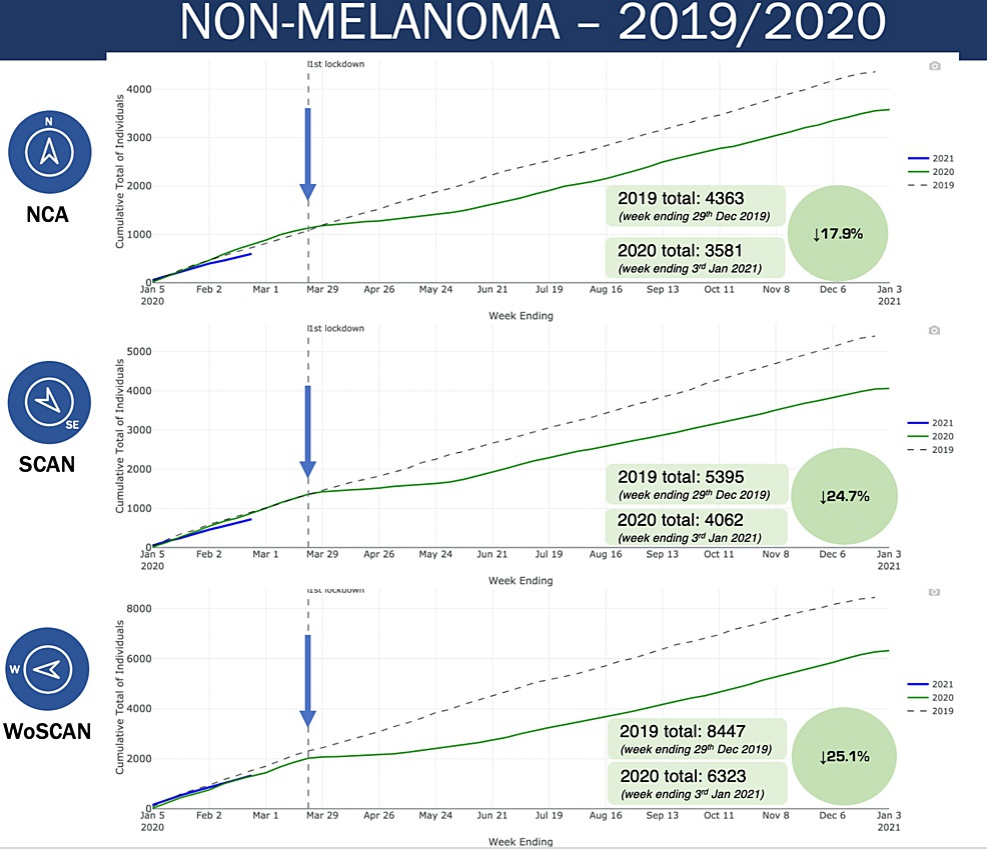
Non-melanoma skin cancer diagnoses in NCA, SCAN and WoSCAN networks NCA: North Cancer Alliance, SCAN: South East Scotland Cancer Network, WoSCAN: West of Scotland Cancer Network

**Figure 4:**
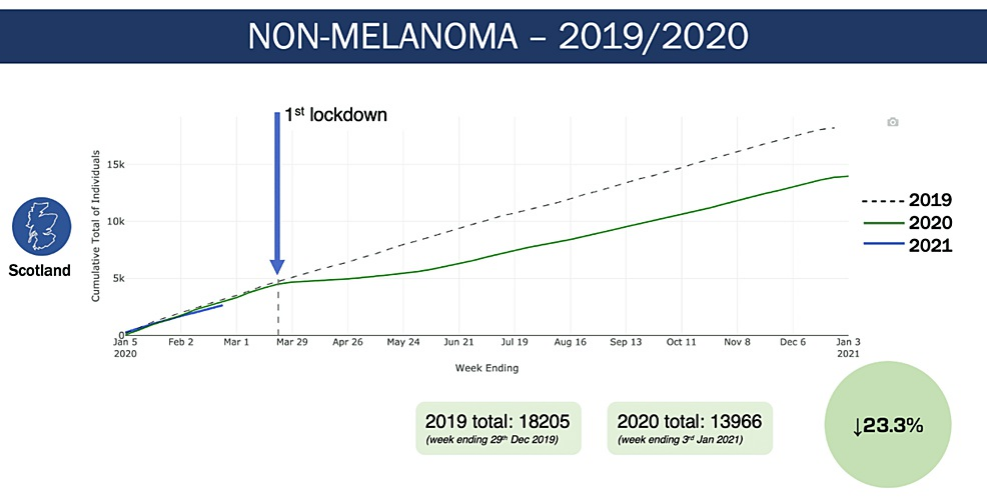
Non-melanoma skin cancer diagnoses in all of Scotland

Data for non-melanoma skin cancer for all three cancer networks show a similar divergence pattern in 2020, with a 17.9%, 24.7% and 25.1% reduction in non-melanoma skin cancer diagnoses for the NCA, SCAN and WoSCAN networks respectively (Figure 3). In all of Scotland there was a 23.3% reduction in non-melanoma skin cancer diagnoses in 2020 when compared to 2019 (Figure 4).

**Figure 5:**
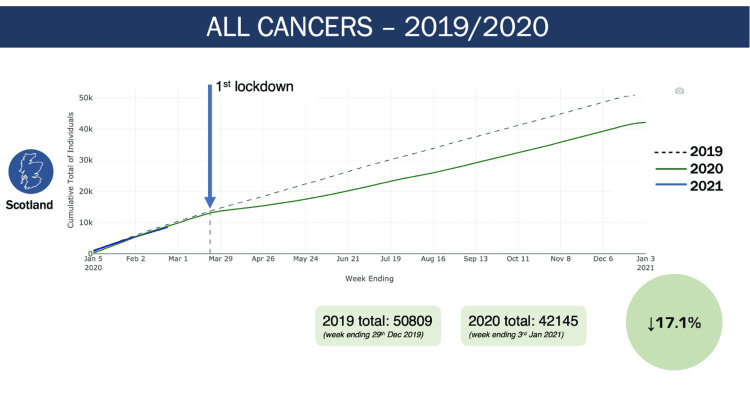
All cancer diagnoses in all of Scotland

This is a trend which is not unique to skin cancer, and if we look at all cancer diagnoses in 2020, compared to 2019 (Figure 5), we again see a divergence shortly after the first lockdown in 2020, which led to an overall 17.1% reduction in all cancer diagnoses.

## Discussion

It is important to note that the number of skin cancer diagnoses is case identification which is not synonymous with incidence or prevalence of a disease, which will continue irrespective of the COVID-19 pandemic. It is for this reason that we are concerned about “missing” cases which are out there in the community and have not made themselves known to healthcare professionals as a result of the pandemic.

The reason this is of concern, in melanoma for example, is because we know that delay in diagnosis leads to more advanced disease at presentation which can in turn lead to poorer outcomes. A systematic review by Neal et al. has found eight studies showing positive association between shorter interval to diagnosis and favourable outcomes in melanoma [3]. Meanwhile data from Office for National Statistics shows that stage 1 melanoma has a five-year survival of 99.6%, which decreases to 80.4% and 70.6% for stages 2 and 3 respectively [4].

The other concern is that a backlog of cases will create extra challenges down the line as more advanced cases come through and extra resources will be needed to treat them. To put this into perspective, the current number of patients waiting for treatment in the NHS sits at a record high of 5.98 million and continues to grow [5].

When thinking about reasons for the above data trends, we know we are facing challenges at both primary and secondary care levels. From a primary care perspective, we have seen a significant drop in urgent suspected cancer referrals shortly after the first lockdown [6]. April 2020 has seen a 60% decrease in referrals compared to 2019; we propose a number of important reasons for these statistics [6].

First of all, we know that there have been strong “stay at home” messages during the pandemic with some patients not seeing healthcare professionals due to a fear of contracting or transmitting COVID-19 [7]. Just as in secondary care, we have also seen a significant reduction in face-to-face appointments in primary care leading to a perceived or actual inability for patients to see their general practitioner [8]. With telemedicine playing a vital role in maintaining services during the pandemic, the elderly population (who make up a large proportion of skin cancer patients) may not be as technologically adept at utilising this resource [9]. Another barrier may be patients’ perception of wasting doctors’ time, with some patients prioritising a skin lesion differently to red flag signs of other cancers [10-11]. This is reflected in the data which shows a greater reduction in skin cancer diagnoses compared to all cancers in general. Loss of opportunistic pick-ups during routine appointments is another factor [12]. This has been exacerbated by the fact that social isolation in the elderly has led to them not seeing their relatives who may normally point out a skin lesion that may be of concern.

At the secondary care level, we have seen a reduction in theatre and clinic capacity as well as reduction in capacity of our pathology departments, with staff sickness and isolation continuing to play a role.

To address these issues we have identified a number of potential solutions. With Scotland being a diverse country, it may be advantageous to implement these as bespoke local solutions at the NHS board level to address the unique needs of local communities. Educating patients to be on the lookout for any suspicious skin lesions via public health campaigns can be an effective strategy to increase awareness and subsequent presentation of patients to their general practitioner, which may prompt an onward referral to a specialist. Likewise it is important to spread the message amongst other healthcare and allied professionals to highlight reduction in skin cancer diagnoses, so that they can also be on the lookout, potentially doing a skin check during routine appointments with patients. Increasing face-to-face appointment capacity and improving telemedicine accessibility are some of the strategies that can be implemented to increase the likelihood of a suspicious lesion being picked up during routine appointments. With us facing a scarcity of pathologists, using digital technology and bringing in a national reporting system for central processing and reporting of skin cancer cases could help transform the service [13]. The benefits of digital transformation of pathology services include research information being gathered with ease, prompt electronic referral and consultation via email and image transmission, and digital external quality assurance [13].

There are limitations to the data presented in this article. We know that there are multiple factors at play responsible for the progression and presentation of patients with cancer, and it is difficult to know how much of the drop in the number of cases has been influenced by the COVID-19 lockdown as this data does not consider other confounding factors. We are able to hypothesise the likely factors behind the drop in the number of diagnoses however the solutions proposed are not straightforward and are time-consuming to implement. Data seen in 2021/2022 suggest that we are now back to the expected number of skin cancer diagnoses, however this still does not make up for the “missing cases” during the pandemic. It may be that we are now seeing more advanced cases “missed” during the pandemic, however are still not seeing the expected number of “early” cases. The reasons for this are likely to be multifactorial and these are not reflected in the data.

## Conclusions

We have seen a notable reduction in skin cancer diagnoses in 2020 compared to 2019 (pre-pandemic), a trend seen across all areas of Scotland, as well as for all cancers in general. Changes in health-seeking behaviours, as well as availability of and access to services, have had a significant contribution to this. We need urgent interventions to mitigate the effects of the COVID-19 pandemic on probable patients with skin cancer, however the reasons for the reduced number of cancer diagnoses are likely to be multifactorial and unique solutions at the local health board level are required in order to solve this issue. Further research into this area is also needed to look at confounding factors that may be impacting the reduced number of cancer diagnoses.
